# Perspective Insights into Disease Progression, Diagnostics, and Therapeutic Approaches in Alzheimer's Disease: A Judicious Update

**DOI:** 10.3389/fnagi.2017.00356

**Published:** 2017-11-01

**Authors:** Arif Tasleem Jan, Mudsser Azam, Safikur Rahman, Angham M. S. Almigeiti, Duk Hwan Choi, Eun Ju Lee, Qazi Mohd Rizwanul Haq, Inho Choi

**Affiliations:** ^1^Department of Medical Biotechnology, Yeungnam University, Gyeongsan, South Korea; ^2^Department of Biosciences, Jamia Millia Islamia, New Delhi, India

**Keywords:** Alzheimer's disease, diagnostics, drugs, neurodegeneration, therapeutics

## Abstract

Alzheimer's disease (AD) is a neurodegenerative disorder characterized by the progressive accumulation of β-amyloid fibrils and abnormal tau proteins in and outside of neurons. Representing a common form of dementia, aggravation of AD with age increases the morbidity rate among the elderly. Although, mutations in the ApoE4 act as potent risk factors for sporadic AD, familial AD arises through malfunctioning of APP, PSEN-1, and−2 genes. AD progresses through accumulation of amyloid plaques (Aβ) and neurofibrillary tangles (NFTs) in brain, which interfere with neuronal communication. Cellular stress that arises through mitochondrial dysfunction, endoplasmic reticulum malfunction, and autophagy contributes significantly to the pathogenesis of AD. With high accuracy in disease diagnostics, Aβ deposition and phosphorylated tau (p-tau) are useful core biomarkers in the cerebrospinal fluid (CSF) of AD patients. Although five drugs are approved for treatment in AD, their failures in achieving complete disease cure has shifted studies toward a series of molecules capable of acting against Aβ and p-tau. Failure of biologics or compounds to cross the blood-brain barrier (BBB) in most cases advocates development of an efficient drug delivery system. Though liposomes and polymeric nanoparticles are widely adopted for drug delivery modules, their use in delivering drugs across the BBB has been overtaken by exosomes, owing to their promising results in reducing disease progression.

## Introduction

Alzheimer's disease (AD) is recognized as a disease of neurons and neuronal circuits. It arises as a result of progressive accumulation of β-amyloid fibrils (β-amyloid plaques) and abnormal forms of tau (tau tangles) within and outside of neurons (Jucker and Walker, 2013; Jaunmuktane et al., 2015). Approximately 46.8 million people over the age of 60 years have been diagnosed with AD worldwide (Prince et al., 2016). Though occurrence of the early onset of dementia is <1% per 4,000 individuals, the projected figure is estimated to be 131.5 million in 2050 (Prince et al., 2016). The projected increase in the prevalence of dementia is substantially higher in developing countries than in the USA and Europe, which already have a high proportion of older individuals in their populations. The Alzheimer's Association presented an estimate of 5.5 million people suffering from AD in the USA, and the incidence of AD in the USA is projected to be 7.7 millions in 2030 and 11–16 millions in 2050 (ADFF, 2016).

The pathology of AD begins well before symptom manifestation, with intracellular accumulation of neurofibrillary tangles that arise via abnormal tau protein phosphorylation and extracellular deposition of Aβ-plaques (Selkoe, 1994, 2001a,b). Interfering with neuronal communication, deposition of β-amyloid plaques causes neuronal death, while tau tangles Blocks transport of essentials into interior of the neurons. Characterized by progressive memory loss and cognitive impairment, advanced stage AD patients show symptoms ranging from neuron inflammation to neuron death. Various risk factors promote pathological changes well before the onset of clinical symptoms of AD. In addition to cardiovascular risk, studies suggest a significant contribution of lifestyle related factors such as obesity, diabetes, depression, smoking, and insufficient diet in dementia. This review presents an overview of AD with recent updates on epidemiology, factors that aggravate the disease, and a prospective insight into diagnostic markers and therapeutic options for disease treatment.

## Genetic susceptibility

AD represents one of the greatest health-care challenges of the twenty-first century. Its dependence on age and genetic background has resulted in its classification as either familial (FAD; showing early onset), which is observed in 5% of AD cases, or sporadic (SAD; showing late disease onset), which shows a high disease incidence rate. Though ApoE4 is a well-characterized risk factor in SAD, disease etiology in FAD is attributed to mutations of *amyloid precursor protein* (APP), *presenilin*1 (PS1), and *presenilin* 2 (PS2) genes (Goate et al., 1991; Levy-Lahad et al., 1995; Rogaev et al., 1995; Sherrington et al., 1996; Selkoe, 2001b). Accounting for 15% of total ApoE, APOE4 interferes with the clearance of Aβ from brain. Differing in amino acid substitutions at 112 and 158 positions, ApoE4 carries two arginines, while ApoE3 have two cysteines and ApoE2 has arginine and cysteine at these positions (Mahley and Huang, 2009). Attribution of APOE4 to AD is 50% in homozygous (Apo E4/E4) and 20–30% in heterozygous condition with APOE3 (Genin et al., 2011).

Over the time, substantial evidences regarding accumulation of misfolded Aβ and tau tangles (so-called seeds of pathological consequences) in the brain of patients suffering from AD have been established (Karran et al., 2011). Evidential support of Aβ and tau involvement comes from FAD studies that report mutations in APP (Aβ precursor) and PS1&2 (catalytic γ-secretase subunit; Karch and Goate, 2015; Ahmad et al., 2016). Having a critical role in the multi-causality of dementia (Boyle et al., 2013), mutations in APP enhance aggregation, while PSEN1&2 mutations cause less efficient APP processing, leading to longer and more hydrophobic Aβs (Scheuner et al., 1996; Chávez-Gutiérrez et al., 2012; Wong et al., 2013; Table 1). Though mutations in APP and PS1&2 accelerate generation of disease seeds, decreased Aβ clearance (Mawuenyega et al., 2010) and increased Aβ accumulation (Wahlster et al., 2013) enhances SAD.

**Table 1:** Summary of mutations predicted in genes that have been associated with the occurrence of AD.

**S. No**	**Gene**	**No. of mutations**	**Mutation type**	**Representation**	**Location**	**Effect**
1.	APP	52	Point, Missense, Silent, Deletion	Both coding & non-coding	Exon 5 = 1 Exon 6 = 3 Exon 7 = 2 Exon 11 = 2 Exon 12 = 2 Exon 13 = 2 Exon 14 = 4 Exon 16 = 10 Exon 17 = 25 Non-coding = 1	Pathogenic = 1 Not-pathogenic = 15 Unclear pathogenicity = 9 Protective = 1
2.	MAPT	15	Point, Missense, Deletion	Both coding & non-coding	Exon 1 = 2 Exon 3 = 1 Exon 4a = 5 Exon 6 = 1 Exon 7 = 1 Exon 8 = 1 Exon 10 = 2 Non-coding = 1 *In silico* = 1	Pathogenic = 0 Not-pathogenic = 5 Unclear pathogenicity = 10 Risk modifier = 1
3.	PSEN1	241	Point, Missense, Insertion, Deletion, Complex	Both coding & non-coding (Intron)	Exon 4 = 22 Exon 5 = 46 Exon 6 = 27 Exon 7 = 56 Exon 8 = 36 Exon 9 = 7 Exon 10 = 7 Exon 11 = 22 Exon 12 = 17 Intron = 1	Pathogenic = 223 Not-pathogenic = 3 Unclear pathogenicity = 16
4.	PSEN 2	45	Point, Missense, Insertion, Deletion	Coding	Exon 3 = 2 Exon 4 = 8 Exon 5 = 12 Exon 6 = 3 Exon 7 = 11 Exon 8 = 1 Exon 9 = 1 Exon 10 = 3 Exon 11 = 2 Exon 12 = 2	Pathogenic = 18 Not-pathogenic = 12 Unclear pathogenicity = 15

## Mitochondrial stress in AD

Performing vital biochemical functions, mitochondrial dysfunction significantly affects progression in the pathogenesis of AD (Swerdlow et al., 2014). Associated with the regulation of cellular metabolism, functional impairment of metabolic enzymes, in particular enzymes of the TCA cycle, causes reduction in the energy metabolism in brain (Huang et al., 2003; Bubber et al., 2005). Studies of the AD brain have revealed significant impairment in the functioning of pyruvate dehydrogenase complex (PDHC) and α-ketoglutarate dehydrogenase complex (KGDHC) enzymes, followed by impairment of isocitrate dehydrogenase (Huang et al., 2003; Bubber et al., 2005). Although, increased activity of succinate and malate dehydrogenases was observed, activities of the remaining four enzymes remains unaltered. Considering the high energy demand of neurons, a role of mitochondrial oxidative stress leading to energy imbalance appears to have a considerable effect on neurodegeneration.

Mitochondrial dysfunction, observed as altered mitochondrial DNA and increased cytochrome oxidase (COX) levels, indicates oxidative damage to the neurons of AD patients (Hirai et al., 2001; Nunomura et al., 2001; Moreira et al., 2006, 2007, 2009; Su et al., 2008). In concert with the amyloid hypothesis, altered APP processing increases Aβ deposition (Furukawa et al., 1996), whose aggregation causes oxidative stress through an increase in the production of H_2_O_2_ (Readnower et al., 2011). Inhibition of the mitochondrial electron transport chain (ETC) causes increased ROS production, which damages proteins, lipids, and nucleic acids, observed as increases in 8-hydroxy-2-deoxyguanosine (8-OHdG) and nitrotyrosine levels (Wang et al., 2005). Accumulation of Aβ in the synaptic mitochondria makes them susceptible to changes in synaptic Ca^2+^ as they have high levels of cyclophilin D (CypD; Sayre et al., 2005). Being a component of the mitochondrial permeability transition pore (mPTP), CypD translocation from matrix to mPTP increases interaction of CypD-mPTP with adenine nucleotide translocase resulting in opening of the pore and as such collapse of membrane potential, which ultimately leads to neuronal death (Juhaszova et al., 2004). An increase in the oxidative burden of mitochondria mediates activation of FoxO transcription factor (Kops et al., 2002; Fu and Tindall, 2008), which is associated with induction of SOD and catalase activity, as well as causing cell cycle arrest and cell death (Castellani et al., 2002). Increased ROS attenuation of the antioxidant arsenal of mitochondria alters the cellular redox state. Increased ROS levels act as an autophagy trigger and subjects mitochondria to mitophagy (Scherz-Shouval and Elazar, 2011).

## Endoplasmic reticulum (ER) stress in AD

Acting as a site of protein synthesis, disruption in the proteostasis causes accumulation of unfolded proteins in the ER lumen (Wu and Kaufman, 2006; Ron and Walter, 2007). To reduce abnormal protein aggregation, unfolded proteins are translocated to cytoplasm for degradation; a process referred as ER-associated protein degradation (ERAD; Hetz et al., 2011; Walter and Ron, 2011). Attainment of saturation in the ER's protein-folding capacity elicits a dynamic signaling response referred as unfolded protein response (UPR; Lee, 2001; Ledoux et al., 2003). Recognition of unfolded proteins by stress sensors such as inositol requiring protein 1 (IRE1), protein kinase RNA (PKR) like ER kinase (PERK), and activating transcription factor 6 (ATF6) triggers downstream signaling via transcription factors (Hetz and Mollereau, 2014). PERK induces rapid translational attenuation by inhibition of the eukaryotic translational initiation factor 2α (eIF2α). PERK-mediated phosphorylation of eIF2α also favors translation of transcription factor ATF4, which is capable of controlling expression of genes related to amino acid metabolism, autophagy, and apoptosis. Increased phosphorylation of eIF2α in PS1 mutant knockout mice confirmed the inhibition of eIF2α phosphorylation by PS1 (Milhavet et al., 2002). Additionally, PS1-mediated abnormal processing of IRE1 disturbs UPR by interfering with the ER stress. Activation of IRE1 signaling induces splicing of X-box binding protein 1 (XBP-1), which controls the expression of lipid synthesis, ER protein translocation, protein folding, and ERAD genes (Hetz et al., 2011; Walter and Ron, 2011). Polymorphism in the promoter region of XBP-1, which reduces its transcription, is considered as a risk factor for AD (Liu et al., 2013). Expression of GPR78 and GPR94 associated with the refolding of unfolded proteins attenuated in PS2-expressed cells is attributed to impaired IRE1 phosphorylation. Though protection against Aβ toxicity through enforced *Xbp1* expression in a *Drosophila melanogaster* AD model is attributed to reduction in the release of Ca^2+^ from ER (Casas-Tinto et al., 2011), a similar effect in *Caenorhabditis elegans* AD model is correlated with augmented stress levels and enhanced autophagy (Safra et al., 2013). Nuclear translocation of ATF6 following protease cleavage activates ERAD genes and XBP-1. Moreover, ATF4, ATF6, and XBP-1 stimulate C/EBP homologous protein (CHOP) and its target growth arrest and DNA damage inducible 34 (GADD34), as well as pro-apoptotic components of the B cell lymphoma-2 (BCL2) family of proteins (Xu et al., 2005). Neurons expressing p-tau show enhanced UPR activation (Hoozemans et al., 2009; Abisambra et al., 2013). Excessive adaptive capacity of UPR triggers pro-apoptotic events through upregulation of the cell death genes such as caspase-12 (Szegezdi et al., 2003).

## Autophagy in AD

Degradation of non-essential cellular components, such as misfolded and aggregated proteins, occurs through the autophagy-lysosomal system (ALS; Li et al., 2010; Murrow and Debnath, 2013). Activated by oxidative stress, nutrient starvation, etc., clearance of unwanted entities helps in restoring substrates for cellular remodeling (Ichimura and Komatsu, 2010; Li et al., 2010; Murrow and Debnath, 2013). Abundance of growth factors and cellular nutrients activates mTOR kinase, whereas a starvation state exerts inhibitory effect on mTOR kinase activity. The mTOR kinase-mediated phosphorylation of ATG13 prevents its association to Unc-51 like kinase (ULK), and recruitment of focal adhesion kinase family interacting protein of 200 kD (FIP200) inhibits autophagy, whereas inhibition of mTOR activates phosphatases that cause dephosphorylation of ATG13 (Kundu, 2011; Lee et al., 2012), thereby promotes autophagy. Although mTOR-dependent autophagy is prominent, there are reports of mTOR-independent autophagy mediated by (1) ATG5 and ATG7 via microtubule associated light chain 3-II (LC3-II; Nishida et al., 2009); (2) autophagic proteins Beclin1, Bcl-2, and ULK1 (Nishida et al., 2009; Shimizu et al., 2010); and (3) non-canonical signaling events involving Ca^2+^. In addition, Ca^2+^ has a prominent role in both canonical and non-canonical mTOR-independent autophagy (Cárdenas and Foskett, 2012; Decuypere et al., 2013). Deletion of Beclin-1 in AD mouse models has resulted in increased Aβ accumulation, while its overexpression leads to reduction in amyloid pathology (Pickford et al., 2008; Jaeger and Wyss-Coray, 2010). Though IP3 receptor-mediated Ca^2+^ signaling inhibits autophagy, an increase in the cytosolic Ca^2+^ level promotes autophagy (Criollo et al., 2007; Wang et al., 2008; Khan and Joseph, 2010; Vingtdeux et al., 2010). Some studies have linked PS mutation in FAD with neuronal dysfunction and apoptosis via Ca^2+^ dysfunction (Del Prete et al., 2014; Duggan and McCarthy, 2016).

Despite entrapping non-selective molecules, selective trapping of various molecules occurs through interaction of LC3-II with cargo-receptors such as nuclear dot protein-52 (NDP52), p62, and neighbor of BRAC1 (NRB1; Bjørkøy et al., 2005; Kirkin et al., 2009; Jo et al., 2014). Acting as an autophagic adaptor for degradation of toll/IL-1 receptor homology domain containing adaptor including IFN-β (TRIF) and tumor receptor associated factor-6 (TRAF6), a role of NDP-52 in docking of autophagosomes to T6BP, myosin VI, and optineurin for maturation has been reported (Inomata et al., 2012; Tumbarello et al., 2012). Interaction of NBR1 and p62 with ubiquitinated misfolded proteins and members of the ATG8 family makes them critical for autophagosome formation (Kabeya et al., 2000; Bjørkøy et al., 2005; Pankiv et al., 2007; Kirkin et al., 2009). PS1 mutations that cause a loss of lysosomal acidification ultimately lead to loss of lysosomal proteolytic activity (Lee et al., 2010). Disruption in the lysosomal function causes accumulation of autophagosomes containing protein aggregates, as observed in the AD brain. Studies indicate involvement of NDP-52 as a downstream facilitator of nuclear factor erythroid-2 related factor 2 (Nrf2)-mediated tau (phosphorylated) degradation (Jo et al., 2014). Reduction in the autophagy-aggravated AD pathology underlines the critical role of autophagy in the removal of Aβ aggregates (Cai et al., 2012; Di Domenico et al., 2014).

## Biomarkers of AD

Cortical amyloid deposition of Aβ and phosphorylated tau (p-tau) are core biomarkers of AD in CSF (Blennow et al., 2010). With high accuracy diagnostics, specificity of the core repertoire of biomarkers is in the 80–90% range in the mild cognitive impairment stage of AD (Shaw et al., 2009; Visser et al., 2009). Despite this, assessment of the sensitivity of disease biomarkers reflects an imperfect criterion in the gold standard of clinical diagnosis of the disease because it fails to distinguish pathological changes such as plaque count between cognitively normal elderly persons and those with AD (Coart et al., 2015; Curtis et al., 2015). Changes in α-synuclein (α-syn), TDP43, and several vascular indicators, are other pathological markers in patients suffering from AD (Kovacs et al., 2013).

A step to increase the specificity and sensitivity of biomarkers has led to the screening of several different entities for use in the diagnosis of AD. One such biomarker involves Aβ oligomers (toxic forms of Aβ), which are associated with synaptic dysfunction (Overk and Masliah, 2014). Though increases in the Aβ oligomers are observed in AD, their limited occurrence in the CSF hinders their reliability in the diagnosis of AD (Hölttä et al., 2013; Yang et al., 2013). Neurogranin, a dendritic protein, is another synaptic biomarker candidate (Díez-Guerra, 2010). Increased amount of neurogranin in CSF of AD patients is correlated with the progression of mild cognitive impairment (Kvartsberg et al., 2015). Additionally, an increase in the level of presynaptic protein SNAP25 might indicate AD diagnosis (Brinkmalm et al., 2014). Of the other biomarkers, screening of CNS-specific protein candidates in blood also seems suitable; when sampling populations for AD. Combinations of proteins, lipids, and other molecules have been used in the assessment of AD (Henriksen et al., 2014; Mapstone et al., 2014). However, as they may be secreted in low amounts and are prone to degradation by plasma proteases, their suitability as biomarkers for AD is uncertain. Similarly, tau-imaging, which is commonly employed in drug trials to determine delay in the progression of AD may be another potentially suitable option for AD diagnosis.

## Therapeutic approaches to combat AD

The fact that critical care provides patients a better quality of life family care is considered as the mainstay in the treatment of AD. At present, efforts are being made on two fronts; toward developing disease modifying therapies that can uphold progression of the disease, and; developing drugs that can act as disease pathway blockers. As Aβ and tau hyper-phosphorylation are well-recognized hallmarks of AD (Lee et al., 2005; Querfurth and Laferla, 2010; Ahmad et al., 2017), current drug approaches are aimed at:

### Improved APP processing via inhibition and/or activation of enzymatic machinery

To date, only five drugs have been approved by the USA FDA for treatment of cognitive manifestations of AD (Table 2). Of the different drug regimes, glutamate agonist memantine, and acetylcholine esterase (AchE) inhibitors such as rivastigmine, donepezil, and galantamine are employed in the treatment of the dementia phase of AD (Raina et al., 2008). Blocking the pathological stimulation of NMDA receptors, memantine protects neural cells from glutamate-mediated excitoxicity. AchE inhibitors cause temporary slowdown in cognitive function by decreasing activity of cholinesterase, leading to enhanced acetylcholine (Ach) levels and, thereby, brain functions (Godyn et al., 2016). As disruption of AchE has a direct correlation with NFT and Aβ deposits (Tavitian et al., 1993), AchE inhibitors stabilize cognitive performance and daily functioning in early dementia stages, whereas application of memantine provides benefits to patients suffering from moderate to severe dementia (Godyn et al., 2016). The fifth drug, tacrine is less prescribed due to its hepatotoxicity. Although these drugs provide symptomatic improvement in AD, benefits are generally short lived and with no effect on the pathogenic mechanism or on disease progression. Research into potential strategies is currently directed at screening bioactive compounds for their effect on enzyme inhibition, aggregation, prevention of Aβ formation, and upregulation in the removal of toxic Aβ.

**Table 2:** Summary of therapeutic options available in the treatment of AD.

**S. No**	**Therapy type (categorization)**	**Phase 1/2**	**Phase 3**	**Phase 4**		**Approval for treatment**			
		**Number**	**Name**	**Name (synonyms)**	**Target**	**Name**	**Synonyms**	**Target**	**Approved for**
1.	Small molecules	50	LMTM, AGB101, ALZT-OP1, AVP-786, AZD3293, Aripiprazole, Azeliragon, Brexpiprazole, Elenbecestat, ITI-007, Idalopirdine, Intepirdine, Masitinib, Nilvadipine, Pioglitazone, Verubecestat	AVP-923 (Nuedexta, Zenvia)	Other Neurotransmitters	Donepezil	Aricept™, Donepezil hydrochloride, Eranz®, E 2020	Cholinergic system	Alzheimer's disease
				Carvedilol (Coreg, Artist, Aucardic, Dilatrend, Kredex)	Unknown	Galantamine	Razadyne™, Reminyl™, Nivalin®	Cholinergic system	Mild to Moderate Alzheimer's disease
				Prazosin (Prazosin hydrochloride, Minipress, VasoflexHypovase,)	Other Neurotransmitters	Memantine	Ebixa™, Memary®, Axura®, Akatinol®, Namenda™,	Other neurotransmitters	Alzheimer's disease
				Resveratrol (trans-3,4′,5-trihydroxystilbene)	Other	Rivastigmine	Exelon™, Rivastigmine tartrate, Rivastach® Patch, Prometax®, SDZ ENA 713	Cholinergic system	Mild to moderate Alzheimer's disease
				Simvastatin (Zocor®, Denan®, Lipovas® Lipex®)	Cholesterol	Tacrine	Cognex™	Cholinergic system	Alzheimer's disease
2.	Dietary Supplement	4	Alpha-Tocopherol (Vitamin E)	Docosahexaenoic acid (DHA; Omega 3 fatty acid)	Others (reduction of amyloid, tau)	–	–	–	–
				Ketasyn (Axona, Caprylic Acid, AC-1202)	Others	–	–	–	–
				Resveratrol (trans-3,4′,5-trihydroxystilbene)	Others (prevention of amyloid deposition, induction of sirtuin-1 gene)	–	–	–	–
3.	Immunotherapy (active)	8	–	–	–	–	–	–	–
4.	Immunotherapy (passive)	11	Aducanumab (BIIB037), Crenezumab (MABT5102A, RG7412), Gantenerumab (RO4909832, RG1450), Solanezumab (LY2062430)	–	–	–	–	–	–
5.	Procedural intervention	2	Continuous Positive Airway Pressure (CPAP)	–	–	–	–	–	–

http://www.alzforum.org/therapeutics

### Immunotherapeutic approaches to existing amyloids

Currently, the most attractive Aβ-directed approach, immunotherapy enlists both active (immune stimulation by vaccine for antibody production) and passive (injection of pre-prepared antibodies) immunization. Engaging both the cellular and humoral immune systems, active immunization is cost effective and ensures long-term high antibody titers. An active vaccination journey begins with AN1792, a full-length Aβ_1−42_ peptide injected with the immune stimulant adjuvant QS21 (Panza et al., 2014). However, on observing meningoencephalitis among 6% of the enrolled AD patients, the phase II AN1792 trial was halted in 2002. Taking strong cognizance of AN1792 trials, larger studies were directed at testing passive immunotherapy by using human anti-Aβ monoclonal antibodies. At present various 2nd generation active Aβ vaccines are undergoing clinical trials; of particular note are CAD106 (Aβ_1−6_; Novartis), ACC001 (Aβ_1−6_; Janssen and Pfizer), ACI-24, V-950, and AD-02 (Aβ_1−6_; Alzforum, 2016). Compared to active immunization that leads to a polyclonal antibody response, target specificity (specific against monomeric Aβ, Aβ fibrils, and their carrier and transport proteins) in passive immunization has advantages both in preventing Aβ-aggregation and in promoting Aβ-clearance in anti-Aβ immunotherapy. The first monoclonal antibody was bapineuzumab directed against Aβ_1−5_ (Abushouk et al., 2017; Xing et al., 2017). Although it reduced Aβ in brain during phase II trials, it failed to achieve significant benefit in a phase III trial, leading to its termination in 2012. This was followed by trials of mAb m266, which showed enhanced Aβ clearance from brain to blood (Demattos et al., 2001; Dodart et al., 2002). Using am266 precursor, Solanezumab directed against mid-domain Aβ_16−24_ revealed dose-dependent increases in plasma Aβ, suggesting cleavage of insoluble species from senile plaques (Farlow et al., 2012; Han and Mook-Jung, 2014). Other Abs undergoing clinical trials include gantenerumab (showing binding specificity for Aβ plaques), crenezumab IgG4 mAb (showing binding specificity for Aβ oligomers, plaques, and fibrils that inhibit aggregation), GSK933766, and BAN2401 mAb (Alzforum, 2016). Reduction in Aβ production is also achieved by using inhibitors against secretases such as NIC5-15, Bryostatin-1, AZD3293, MK8931, and E2609 (Alzforum, 2016).

### Tau centric therapies

Tau centric therapies include the use of putative tau kinase inhibitors, microtubule stabilizers, and tau immunotherapy. Glycogen synthase kinase-3β (GSK-3β), the primary enzyme involved in tau phosphorylation, is considered the primary target for disease modification (Jaworski et al., 2011). As Aβ promotes GSK-3β activity, studies of GSK-3β inhibitors such as tideglusib and AZD1080 were pursued (King et al., 2014; Lovestone et al., 2015). Clinical trials of AZD1080 revealed nephrotoxicity (Eldar-Finkelman and Martinez, 2011), while the trials for tideglusib showed diminished clinical benefits. Microtubule stabilizers inhibit tau aggregation (Bulic et al., 2010). LMTC, a methylthionium chloride (MTC) derivative prevents tau interactions, thereby facilitating its clearance from the brain (Wischik et al., 2015). Other microtubule stabilizers include TPI207 and BMS241027 (epothilone-D). Immunotherapy for tau protein is directed toward prevention of NFT formation. Both AADvac1 (a synthetic tau derived peptide) and ACI-35 (liposome formulated based tau protein) are currently being evaluated for their effects on avoiding tau aggregation (Alzforum, 2016).

### Therapies to combat oxidative stress

Oxidative stress due to reactive oxygen species (ROS), being a major player in neurodegeneration strategies, are currently being devised to combat its emergence at mitochondria (Federico et al., 2012; Yan et al., 2013; Kim et al., 2015). On the forefront, ferulic acid (FA), epigallocatechin-3-gallate, and nano formulation of naturally occurring curcumin were found exerting strong antioxidant, anti-inflammatory and amyloid disintegration properties (Rezai-Zadeh et al., 2005; Yang et al., 2005; Cheng et al., 2013; Sgarbossa et al., 2015; Cascella et al., 2017). Alleviating mitochondrial dysfunction under diseased conditions, peptide based strategies complemented with different drug molecules have shown positive results in overcoming the inefficiency of low antioxidant levels (Kumar and Singh, 2015). Of them, Szeto-Schiller (SS) peptides displaying a sequence motif that directs its accumulation inside mitochondria inhibit lipid peroxidation. Accumulation of SS31 on inner mitochondrial membrane prevents release of cyt-C (Szeto, 2008; Kumar and Singh, 2015). Similarly, accumulation of drugs such as MitoQ inside mitochondria increases its potential to neutralize free radicals several 100-folds than that attributed by natural antioxidants (Tauskela, 2007; Ross et al., 2008). Probucol, a drug that helps establishment of balance of mitochondal fission-fusion processes, also maintains AD mitochondria induced extracellular signal regulated kinase (ERK) activation (Champagne et al., 2003; Gan et al., 2014).

### Autophagy enhancer therapy

As accumulation of the aggregated proteins enhances neurodegeneration, enhancement in the degenerative capacity via, autophagy inducers seems a good therapeutic approach. Their mode of action involves prevention in the accumulation of Aβ plaques and tau tangles via degradation of the aggregates. Of the available drugs, rapamycin acting as mTOR inhibitor was found ameliorating Aβ and tau pathies in AD mouse model (Caccamo et al., 2010; Rubinsztein et al., 2012). Latrepirdine stimulation of autophagy reduces Aβ neuropathy in mouse brain (Steele and Gandy, 2013). Metformin, a PP2A agonist, inhibition of TORC1 prevents hyperphosphorylation of tau protein (Kickstein et al., 2010). Other authophagy enhancers include reservatol and its analogs, RSVA314 and RSVA405, virally packaged BECN1 and its mimetics, nicotinamide, etc (Vingtdeux et al., 2010; Shoji-Kawata et al., 2013). Though, Beclin1 deficiency increases APP and Aβ levels, its over-expression in cultured neurons cause significant reduction in Aβ accumulation (Pickford et al., 2008). Additionally, PS1 and PS2 serving as a catalytic subunit of γ-secretase was found acting as autophagy modulators (Lee et al., 2010). Associated with the substrate cleavage, their knockdown was found exhibiting inefficiency in the clearance of protein aggregates. Cells deficient in PS1 was found having reduced levels of cargo shuttle protein p62, associated with the degradation of abnormal tau (Tung et al., 2014; Caccamo et al., 2017).

Failure of biologics that target amyloids to cross the blood-brain barrier (BBB) indicates the necessity of development of an efficient drug delivery system. Liposomes and polymeric nanoparticles have shown promising results in delivering drugs and other therapeutic molecules (Ha et al., 2016); however, their use as a means to deliver drugs across the BBB has encountered roadblocks associated with biocompatibility and long-term safety. Persistent problems with low biocompatibility, restricted immune escape, circulation stability, and toxicity have led to a gradual shift of research toward the use of exosomes. Their long circulatory half-life, host biocompatibility, target-specific drug delivery ability, and low toxicity have increased the interest in using exosomes to deliver drugs in several neurodegenerative diseases (Lai and Breakefield, 2012; Liu et al., 2016). As drug delivery through exosomes has shown promising results in the ongoing studies, their utilization in reducing disease progression may indicate their suitability in reducing progression of AD.

## Conclusion

AD progresses via aggregation and accumulation in the extracellular milieu of amyloid plaques and intraneuronal neurofibrillary tangles produced by p-tau. Although malfunctioning APP, PS-1 &-2 genes are considered the main culprits behind AD, mitochondrial dysfunction, ER stress and mitophagy significantly increase progression of the disease. Current research provides useful information on new targets and their utilization in designing novel inhibitors or drugs as part of attempts to achieve successful treatment of AD. It also studies different entities for employment as potent biomarkers in disease diagnosis and provides information on therapeutic suitability in the treatment of AD. Further studies on the use of exosomes as drug delivery vehicles are needed in order to reveal and reduce safety and ethical concerns. With increases in newly identified targets, studies pertaining to the design of drugs and other potent therapeutic molecules are needed in order to combat the progression of AD.

## Author contributions

IC, QH, and AJ: conceived the idea; AJ and MA: contributed to writing of the manuscript; AJ, SR, AA, DC, and EL: contributed to upgrading the contents and preparing the tables.

### Conflict of interest statement

The authors declare that the research was conducted in the absence of any commercial or financial relationships that could be construed as a potential conflict of interest.
